# Abstracts and Gleanings

**Published:** 1885-05-20

**Authors:** 


### ABSTRACTS AND GLEANINGS.

**The Use and Abuse of the Tampon in Abortion.**—Dr. J. W. Keene read a paper with this title before the Buffalo Obstetrical Society, meeting of March 24, 1885. The President, Dr. W. W. Potter, in the Chair.

Dr. R. L. Banta said that he had not found the danger of inducing abortion by the use of the tampon so great as Dr. Keene and the authors he cited had stated. He uniformly used the tampon if he found a woman losing a thimbleful of bood. Now, if he had plenty of time, he let it flow, but, if he was compelled to leave the patient, he tamponed. Much depended on the judgment of the physician; the older he grew the less he would fear hemorrhage.

Dr. Fredrick believed the indications for the use of the tampon were as stated by Dr. Keene. He considered the colpeurynter preferable to the old-fashioned tampon, and used it in his practice.

Dr. Ingraham said, that if a woman did not flow much, he felt at liberty to go away. He did not think the tampon always produced abortion.

Dr. Stockton had been rather frequently called upon to consider whether or not he would use the tampon. In the paper it had been pretty well stated when it should be used ; but the question of using the tampon occurred at other times than those mentioned, for instance, after the escape of the fœtus, when there was retention of the secundines.

Dr. Hartwig would say that, if the tampon was to be used, the indications for its use had been correctly given. He was of the opinion that the tampon should be given up almost entirely. He had used it in some cases. He fully believed, as Schrode stated, that a woman would not bleed to death from abortion before the fourth month. The old-fashioned tampon would certainly excite uterine contractions : and the same might be said of the colpeurynter if sufficiently dilated to check the hemorrhage. There were however, other ways of tamponing, as compressed sponge. Another way was to tampon the cervix through a speculum. He thought he would do the latter if he tamponed at all. But in hemorrhage during pregnancy, and after the expulsion of the child, he had decidedly come to the conclusion that a tampon was unnecessary, because, if the bleeding was slight, the woman could bear it. Small doses of ergot, or even large doses—which never of themselves produced abortion—would check the bleeding. The life or death of the child ought to be decided with a good deal of probability. If the woman had bled much, and the cervix was patulous, we could be sure that the baby was dead. As a scientific ruling, he would put forth the idea that the tampon should not be used at all. Its real value consisted in exciting uterine contractions.

Dr. Tremaine was very glad to hear these views expressed. He did not practice obstetrics, except in consultation. Some years ago he used to do a great deal of midwifery. He never used a tampon but once, and never would again if he could help it. He thought it one of the most barbarous, unsurgical, and nonsensical things ever invented. Dr. Hartwig had expressed nearly his own ideas. He used to see much hemorrhage, and was accustomed to control it by injecting alum-water into the uterus. He had never seen a death from post-partum hemorrhage. When there was much bleeding, abortion was sure to take place. About the surest way to bring it on was to give opium.

Dr. Lothrop said that how these gentlemen practiced obstetrics and dealt with abortion in the second and third months without the use of the tampon he did not understand. It was an absolute necessity. He very seldom used it unless the life of the embryo was destroyed. How these gentlemen could see the woman lose such a large amount of blood when they could stop it with a plug he did not understand. When he had been compelled to use the tampon it had almost always been from retention of the secundines after the expulsion of the fœtus. He could not get along successfully in abortion without the use of the tampon. His way of using a tampon was to pack the vagina with cotton pledgets by means of a Sims's speculum. As for septic infection following the use of the tampon, it had never occurred in his practice. There were two symptoms he looked upon as important in abortion—pain and hemorrhage ; and the two things he recommended were opium and a tampon.

Dr. Girard had an opinion on this subject, and was willing to give it for what it was worth. He had many cases of hemorrhage of this character, and had tried many means to check it. When he found the os partly dilated and the cervix softened, he was satisfied of the death of the fœtus, and that the best procedure was to bring on rapid contraction and prevent further hemorrhage. He believed the tampon in nearly every case would bring on abortion; so he would not use it while there was any chance that the fœtus was alive. If the physician was satisfied that the child was dead, he did not see that he could do better than to tampon.

The president said it was difficult to understand the import of the paper. In a general way, he inferred that its bearing was against the use of the tampon. He used the tampon himself, and had never yet used it, in more than twenty-six years, when he regretted it. He used the tampon for two purposes: first, in extreme cases, to save life, and more especially to avoid the loss of blood. He thought the gentlemen made a mistake when they allowed their patients to bleed one ounce. He would say that no man should undertake to practice obstetrics in this day and age and permit his patient to bleed. If he did not succeed in the use of the tampon, it was because he did not use it right. He was certain that in case of a violent hemorrhage the only way to control it was to put the patient in Sims's position and pack the vagina so tight that there could be no hemorrhage. If he was called to a case of threatened abortion, and found the os open and the fœtus not accessible, he would not commence by tamponing if he lived near by ; but, if he lived ten miles away, he would tampon the woman for her own safety, and he would be pretty sure that, on removing the tampon the next day, the fœtus and secundines would drop into his hands. After the escape of the fœtus and retention of the secundines, his practice would be to remove them if he could. If he could not do so with tolerable ease, he would tampon. He would not allow the patient to suffer the loss of four ounces of blood. In reference to ergot, gentlemen might give it, if they pleased, in threatened abortion with a hope that it would prevent hemorrhage; but they should not rely on it. He had come almost to think it nearly useless. He did believe in the use of the curette in removing detritus, and then brushing over the cavity with Churchill's tincture of iodine; then, if there was any danger of hemorrhage, the vagina should be tamponed.—*N. Y. Med. Jour.*

**Anæsthetic Agents---The State of the Case.**—During the past year Dr. B. A. Watson, of Jersey City, has been engaged in an experimental study of certain anæsthetics, an account of which was laid before the last meeting of the American Surgical Association, and now reaches us in the form of a reprint from the Transactions of that body. This paper, together with the report of the discussion which followed its reading before the Association, give a very clear idea of the present attitude of the mind of the profession towads the question: "Which is the safest anæsthetic?" The anææsthetics experimented with by Dr. Watson were bromide of ethyl, chloroform and sulphuric ether. The subjects were eighty-two rabbits and twenty-five dogs. These animals were kept in a condition of profound anæsthesia for two hours, and during this time observations were made upon the pulse, respiration and temperature at intervals of about a half an hour. Similar observations were made during the seventy-two hours after recovery from the state of anæsthesia. The experiments showed: 1st. That chloroform is a more depressing agent than ether. 2nd. That a resort to artificial respiration became necessary most frequently during the use of ethyl bromide, less frequently during the use of chloroform, and was never necessary during the use of ether. 3rd. That the death rate by ether was relatively very small. (Rabbits 16⅔ per cent., 62½ by chloroform, and 40 per cent. by ethyl bromide; the mixtures of alcohol, chloroform and ethyl being almost, if not quite, as deadly. Dogs, by ether none died; by chloroform none; by ethyl bromide all died.) 4th. That ethyl bromide, whether given pure or in combination with other anæsthetics, frequently causes more deaths within the forty-eight-hours which follow its administration than during the period in which complete anæsthesia is maintained, especially when the latter has been protracted; although sulphuric ether, chloroform, and their mixtures are generally free from this danger. 5th. That death was invariably preceded by very rapid respirations, regardless of the anæsthetic which produced it. That death occuring during the administration of the ethyl bromide is, in all probability, caused by its paralyizing action on the heart.

Dr. Dawson, of Cincinnati, who opened the discussion, cited two cases from his own practice where death had occurred from heart failure under chloroform. In one case the field of operation

*thrice* became suddenly bloodless ; the third time the patient died. He believes that chloroform is a dangerous agent, killing beyond resuscitation by paralyzing the heart, and that the danger does not depend upon the amount of chloroform administered, or the length of time the patient is kept under the influence, but upon the condition of the patient at the very time of its administration. In support he quoted the case of a Mrs. G., who had taken chloroform in eight confinements, in one of them having been kept under its influence for twelve consecutive hours, and who seated in a dentist's chair, having taken (from the hand of her physician,) half a dozen whiffs of chloroform died of cardiac paralysis. On the other hand Dr. Donald McLean, of Detroit, quotes Simpson's celebrated case of the patient who died under the touch of the knife before chloroform had been administered, and brings forward a similar case of his own. Dr. McLean has been using chloroform liberally for twenty-five years, and has yet to see an accident. Dr. Wm. Byrd, of Quincy, Ill., said that he had lost one patient by chloroform. He had kept her under the influence of the drug on a previous occasion for an hour. "She only breathed a few times after the chloroform was administered when she died." Dr. B. uses a mixture of ethyl bromide, chloroform and alcohol in Johnson's inhaler and finds it safe and agreeable. Dr. L. McLane Tiffany, of Baltimore, spoke in favor of ethyl bromide. He thinks it as safe as any anæsthetic can be in the nature of things, provided that the patient is in the recumbent posture, that the duration of the state of anæsthesia is short and the administration is not repeated at the sitting. Dr. Conner, of Cincinnati, has had one death by chloroform. He feels bound to use ether because it is so much the safer.

From all this we gather that the minds of our ablest physicians are still in an unsettled state upon this most important question ; that bromide of ethyl is an anæesthetic of the use of which we had best be chary for the present, and that it should never be used to produce more than a few moments unconsciousness ; that ether should be regarded as safer than chloroform, but that the latter is as much more dangerous than the former as experiments upon the lower animals would lead us to believe, is not borne out by a vast clinical experience. In considering this question we must remember that chloroform is still *the* anæsthetic throughout most parts of the world, and that its dangers bear a most pertinent relation to the intelligence and attention of the administrator. This matter of the relative safety of chloroform and ether presses upon the whole profesion for solution. It can never be settled. it seems to us, until a number of medical men be found devoted and painstaiking enough to keep a record of every case in which they administer, or assist in administering, either agent and the result, and after many years to give the figures to the world. Then will we be able to determine the percentage of deaths by chloroform, and by ether, and therefore the degree of danger attending the use of either anæsthetic. Then, and not till then, will we know the *true* state of the case.—*N. O. Medical and Surgical Journal.*

**Sodium Salicylate in Neuralgic Headache.**—Since the publication in these columns of a translation of the experience of Dr. Delczersky with sylicate of sodium in neuralgic headache (*National Druggist*, Vol. V. p. 130), I have had the opportunity of testing the remedy in a number of cases, three of which might be termed typical—or rather crucial—the latter word expressing not only the nature of the test, but the sufferings of the patient as well. A history of one of them will suffice for the balance. The patient, aged thirty-nine years, married, and the mother of four children, resident at Mobile, had been subject to severe neuralgic headaches for fifteen years past. Come on first about once a month, these headaches gradually grew more frequent and severe, until at last she spent more than half of her time in bed, her sufferings being described by herself and the attendant physician, as something dreadful to contemplate. Every remedy ever suggested for the relief of neuralgia and headache was tried, almost without effect. After reading Delczersky's article (in the *Midiz. Obozrenyi*), I determined to try sodium salicylate, and sent her by mail the following prescription: Sodium salicylate, 2 drachams; divide into 16 capsules, of which let the patient take 2 when threatened with an attack, and repeat the dose every half hour, until pain is relieved, or until four doses are taken. Two weeks later the patient wrote me: "How can I thank you sufficiently for that prescription? The day it arrived I was in bed with one of my dreadful attacks. I had the medicine prepared and commenced taking it. With the second dose I felt great relief, and by the time I had taken the third all pain had ceased. Since then the trouble has threatened to return several times, but a couple of capsules averted it in each instance." Two months later she writes: "That remedy has cured my headaches. I have taken, as you directed, 2 capsules every morning, and there has been absolutely no return of my headaches. Once or twice when I have been very tired there was an uneasy feeling about the head, but a couple of capsules removed it. I have gained thirty pounds—all the flesh which I had lost, and am in perfect health."

The other cases were similar to this in every respect, and I think that the profession may safely rely on sodium salicylate as being "almost a specific" in this most distressing and hitherto intractable of maladies.

The salicylate may be given in solution or dry, inclosed in capsules. The dose for an adult should not be less than 15 grains repeated every half hour until 60 grains are taken, unless sooner relieved. In cases where the disease is of long standing, it is well to have the remedy continued in daily doses of not less than 15 grs., which should be taken in the morning or on an empty stomach. Of course due attention must be paid to the bowels. Where there is habitual constipation—which will be in three-fourths of the cases where the patients are women—aloin will be found invaluable. Podophyllin will also be found to act admirably, especially where there is a malarial taint. Where the disease is undoubtedly of malarial origin, occasional resort should be had to mild chloride of mercury.—*National Druggist.*

**Hypophosphite of Lime in Cancer.**—Dr. J. B. Johnson, of Washington, D. C., writes as follows in the *Medical and Surgical Reporter:*

Some time ago I received a copy of a lecture by Dr. Hunter McGuire, of Richmond, Va., on the subject of "Cancer of the Breast," in which he recommended the use of hypophosphites of lime and soda. The formula is:

℞. Hypophosphite of lime and soda............... ℥ ss.

Diluted phosphoric acid........................ ʒ ss.

Distilled water.................................. ℥ viij.

M. S.—Teaspoonful in water three times a day, and when indicated. He sometimes uses in addition arsenic and iron, in the forms of chlorides of arsenic and iron.

At the time of reading the lecture I had under my care two cases of cancer: one of the breast and one of the ear, at the angle of the left jaw. About a year before, I was consulted in the case of cancer of the breast; the breast had been entirely excised, but the wound made no effort to heal, and grew to be an ulcer two inches wide by two inches long. The cancer of the ear also presented an ulcer irregular in shape, covering the space of an inch or more in extent. I gave at once internally:

℞. Hypophosphite of lime........................ ʒ iss.

Bromide of potassium.......................... ʒ ij.

Fowler's solution.............................. ʒ iss.

Distilled water................................. ℥ viij.

M. S.—Dose, a tablespoonful every three hours.

And as an external application the following:

℞. Tar,
Alcohol.......................................... ℥ j.

M. S.—Apply freely to the ulcers three times a day.

Both patients have been using the above prescriptions for six months, and the progress of the cancers is not only arrested, but the ulcers almost healed. There is no doubt that the progress of cancer can be delayed by the use of the hypophosphites in combination with arsenic.—*Drug. Cir.*

**Precautions to be Adopted in the Treatment of Phthisis.**—At the International Congress of Hygiene held at La Haye in September last, the following conclusions were adopted regarding the treatment of phthisical patients, after an interesting discussion upon a report presented by Prof. Sormani, of Pavia.

"It is demonstrated that pulmonary phthisis can be, in certain cases, transmitted from the sick to individuals in health. Although the chances of this transmission are limited, prudence requires certain precautions.

"1. No one should be allowed to share the sleeping chamber or bed of a tubercular patient in an advanced stage of the disease. The apartment of a phthisical individual should be constantly aired and ventilated.

"2. The danger resides especially in the sputa, which should not be allowed to go on the floor or on clothing, where they may dry and become converted into dust.

"3. The sleeping-rooms, the bed-linen, and clothing which have been used by consumptives should always be disinfected Steam (at 100° C.) and washing in boiling water are the best means of disinfection.

"4. Convalescents from chest-disorders and feeble and exhausted patients should especially avoid prolonged contact with the tuberculous."—*Revue de Therapeutique Med.-Chir.*, No. 23.

**Action of Iodoform in Diabetes.**—After seeing the effect of iodoform in several cases of diabetes and other diseases, he made experiments which resulted that in doses of 0-05—0.10 the quantity of sugar in the urine, as well as the quantity of urine, considerably decreased; that the number of red blood corpuscles, as well as the hæmoglobin diminished, which is not caused by a destruction of the red blood corpuscles from the evolvement of iodine from the iodoform (the number of blood corpuscles were counted by the hæmometer of Hayem and Nachet, and the hæmoglobin was determined with the chromocytometer of Bizzozero) iodoform has a tendency to lower the arterial blood pressure.—*Bozzolo in Arch. Ital de Biologie. St. Louis Med. Jour.*

**Treatment of Earache.**—M. Moure (Jour. de Med. de Bordeaux, Jour. de Med. et de Chir.) uses habitually a combination of atropia with morphia to quiet the pains of earache, giving relief to otalgia and to cases of subacute otitis of the tympanum and of the Eustachian tube, particularly in children. He prefers a solution of morphia to instillations of oil, laudanum and ether, these substances having the effect of producing a traumatic meningitis and sometimes graver lesions. He prescribes: Sulphate of atropia, 2 to 5 centigrammes; chlorhydrate of morphia, 5 centigrammes; neutral glycerine, 15 grammes; applied on cotton wool at the external auditory meatus, and, when necessary, a drop or two by instillation night and morning into the auditory canal. This will relieve external otitis and furunculous otitis.—*Ibid.*

**Fluoride of Quinine for Enlarged Spleen.**—Fluoride of quinine has recently been recommended by Dr. Weddell, of Calcutta, in the treatment of enlarged spleen. He has investigated the action of fluoric acid and the fluorides, and has come to the conclusion that in cases of chronically enlarged spleens of malarial origin the effects obtained are often very striking. In very small doses the fluorides have produced marked benefit in cases of rickets and other diseases associated with malnutrition of the osseous system. Of the salts of fluoric acid, Dr. Weddell considers those of quinine or quinetum (i. e., of the mixed cinchona alkaloids) to be the best.—*Med. World.*

**Antidote to Strychnine.**—Amyl nitrite is being recommended in various foreign journals as an antidote to strychnine. It may be used either as an injection or by inhalation.—*Exchange.*

**Treatment of Bromidrosis of the Feet.**—Dr. J. S. Stewart, in the *Edinburgh Medical Journal* for March, 1885, recommends as the most satisfactory treatment, to have the feet thoroughly washed in hot water, then steeped for a few minutes in a solution of permanganate of potash of the strength of from 4 to 6 grains in the ounce of water. The feet are then dried, not to be again wetted until complete exfoilation of the tanned cuticle has taken place.

Hebra's lead plaster ointment is then thickly spread on in strips of cloth about one inch and a half broad, and the foot covered from the toes back over the heel as high as the malleoli with these arranged and applied like a sculttetus bandage. Each toe should first be wrapped with a strip of clean rag half an inch broad and thickly spread with the ointment. This dressing should be renewed every twelve hours with fresh rag and ointment, for a period varying from ten to sixteen days, according to the severity of the case and the thickness of the heel skin. In most cases the odor will be very much diminished by the end of the third day, and will not be perceptible by the ninth. The shedding of the skin takes place *pari passu* with the growth of the new cuticle, and may not be completed until the end of the third or fourth week.—*Medical News.*

**Messrs. Wm. R. Warner & Co.'s Concentrated Pepsin** in powder is a preparation much appreciated by all who have occasion to use pepsin in scales, and especially where a concentrated article is required. It forms a clear solution, retains a pulvurulent condition, and mixes with other substances. The use of this preparation in cases of ulcers of various parts of the body—particularly those of the stomach—has given very satisfactory results, from its power of dissolving albuminous products. This pepsin has been found useful as an application to diphtheritic ulcers; also, for indigestion of children, who take it without much trouble. Again, this preparation is found very useful in nervous vomiting and diarrhœa. I find this pepsin has many more times the power of digestion than the saccharated form. The advantages of Warner & Co.'s Pepsin—as, indeed, of all their preparations—are that it is uniform and reliable.—*Ex.*

**Antipyrin.**—This new drug is described as follows: It is in colorless, columnar crystals, or oftener in a voluminous crystalline powder, of a white, or sometimes of a slight reddish color, due to the presence of traces of iron. It is odorless, has a slight bitter taste, and melts at 110° to 130° C. It is very soluble in water, more so in boiling water, and dissolves easily in alcohol or chloroform. With persalts of iron, its aqueous solution is colored red.—*Med. News.*

**Extract of Piscidia.**—Further trial of this preparation as a hypnotic has confirmed the report previously made in its favor. It would appear that in doses of six grains its action is quite prompt, and is unattended by any unpleasant symptoms.—*Med. News.*

**Chloride of Methyl Spray as an Anodyne.**—The following account of the use of chloride of methyl spray, according to the method of Debove, is taken from *L' Union Medicale* for March 3:

M. Tenneson reports thirty-one observations upon the use of the spray of chloride of methyl as an anodyne in various affections. In seven out of ten cases of sciatica the relief was complete; once, pain in the heel existed for somettime after the relief: in two cases only was there a real arrest of the disease. In eleven cases of muscular rheumatism the relief was immediate nine times; in the other two cases a second application of the spray was necessary; the results were equally good in cases of sub-acute and chronic articular rheumatism and the osteo-periosteal pains of the tuberculous; in three cases the spray caused a sudden cessation of pain in the side in lobular pneumonia, and the local refrigeration of 9° F. applied to the chest of a fever patient did not interfere with his spontaneous recovery.

The accidents which may occur are erythema, increased pigmentation, vesication and sloughing. These may be avoided by not prolonging the spraying beyond five or six seconds; even this is too long in regions where the skin is delicate, and perhaps also in women, but upon the latter point M. Tenneson has had no experience, as the cases reported all occurred in men. It is necessary to avoid directing the first jet of spray too perpendicularly against the skin. The erythema may last many weeks, as may also the increased pigmentation. In short, the spray of chloride of methyl is without doubt destined to render a great service against the element of pain; but the end of the subject has not yet been reached.—*Northwestern Lancet.*

**Valoid of Coca.**—The Lancet speaks as follows of this new preparation of coca: "The introduction of a new and reliable preparation of coca will be hailed with satisfaction. The valoid is made from the fresh leaves of the coca plant, each drachm representing the weight of crude drug, including the whole of the alkaloidal and other principles. It has been extensively employed of late, and, curiously enough, is forced to exert a double physiological action. In small doses it acts as a sedative, promoting sleep; while in large quantities, such as three or four drachms, it stimulates the nervous system and induces an increased capacity for mental exertion. It has been used with much success in sleeplessness arising from overwork, worry and anxiety, and also in treatment of impotence, spermatorrhœa, and a number of allied diseases. It has no tonic action:—*Med. News.*

**Evil Effect of Tobacco.**—In a high school in a neighboring city, all the assistant teachers form a league with the teacher of physiology to prevent the use of tobacco by the boys. She is instructed to set before them continually the evils arising from the use of the weed in all forms. She says she makes the deepest impression by showing them how it stunts the growth, and as boys desire nothing so much as to be tall and manly in appearance, many have left off the bad habit for this reason.—*Good Health.*

